# A Text Message Intervention with Adaptive Goal Support to Reduce Alcohol Consumption Among Non-Treatment-Seeking Young Adults: Non-Randomized Clinical Trial with Voluntary Length of Enrollment

**DOI:** 10.2196/mhealth.8530

**Published:** 2018-02-16

**Authors:** Brian Suffoletto, Tammy Chung, Frederick Muench, Peter Monti, Duncan B Clark

**Affiliations:** ^1^ Department of Emergency Medicine University of Pittsburgh Pittsburgh, PA United States; ^2^ Department of Psychiatry University of Pittsburgh Pittsburgh, PA United States; ^3^ Center for Psychiatric Neuroscience The Feinstein Institute for Medical Research Northwell Medical Center Great Neck, NY United States; ^4^ Center for Alcohol and Addiction Studies Brown University Providence, RI United States

**Keywords:** binge drinking, young adult, text messaging

## Abstract

**Background:**

Stand-alone text message–based interventions can reduce binge drinking episodes (≥4 drinks for women and ≥5 drinks for men) among nontreatment-seeking young adults, but may not be optimized. Adaptive text message support could enhance effectiveness by assisting context-specific goal setting and striving, but it remains unknown how to best integrate it into text message interventions.

**Objective:**

The objective of this study was to evaluate young adults’ engagement with a text message intervention, Texting to Reduce Alcohol Consumption 2 (TRAC2), which focuses on reducing weekend alcohol consumption. TRAC2 incorporated preweekend drinking-limit goal-commitment ecological momentary assessments (EMA) tailored to past 2-week alcohol consumption, intraweekend goal reminders, self-efficacy EMA with support tailored to goal confidence, and maximum weekend alcohol consumption EMA with drinking limit goal feedback.

**Methods:**

We enrolled 38 nontreatment-seeking young adults (aged 18 to 25 years) who screened positive for hazardous drinking in an urban emergency department. Following a 2-week text message assessment-only run-in, subjects were given the opportunity to enroll in 4-week intervention blocks. We examined patterns of EMA responses and voluntary re-enrollment. We then examined how goal commitment and goal self-efficacy related to event-level alcohol consumption. Finally, we examined the association of length of TRAC2 exposure with alcohol-related outcomes from baseline to 3-month follow-up.

**Results:**

Among a diverse sample of young adults (56% [28/50] female, 54% [27/50] black, 32% [12/50] college enrolled), response rates to EMA queries were, on average, 82% for the first 4-week intervention block, 75% for the second 4-week block, and 73% for the third 4-week block. In the first 4 weeks of the intervention, drinking limit goal commitment was made 68/71 times it was prompted (96%). The percentage of subjects being prompted to commit to a drinking limit goal above the binge threshold was 52% (15/29) in week 1 and decreased to 0% (0/15) by week 4. Subjects met their goal 130/146 of the times a goal was committed to (89.0%). There were lower rates of goal success when subjects reported lower confidence (score <4) in meeting the goal (76% [32/42 weekends]) compared with that when subjects reported high confidence (98% [56/57 weekends]; P=.001). There were reductions in alcohol consumption from baseline to 3 months, but reductions were not different by length of intervention exposure.

**Conclusions:**

Preliminary evidence suggests that nontreatment-seeking young adults will engage with a text message intervention incorporating self-regulation support features, resulting in high rates of weekend drinking limit goal commitment and goal success.

## Introduction

Young adults have the highest prevalence of hazardous alcohol consumption among all age groups [1], largely due to binge drinking (defined as consuming ≥4 drinks for women or ≥5 drinks for men on any drinking occasion [2]), yet numerous barriers prevent them from seeking help to reduce alcohol consumption [3]. Mobile digital interventions could help provide evidence-based support to young adults who would not otherwise seek help. Systematic reviews suggest that mobile digital interventions can reduce alcohol use in adults [4] and that text message (short message service, SMS) interventions reduce alcohol consumption among young adult populations [5]. Our group has spent the past 6 years iteratively designing and testing a text message alcohol intervention (Texting to Reduce Alcohol Consumption: TRAC), which uses ecological momentary assessments (EMA) to assist self-monitoring, tailor goal support, and provide performance feedback and relevant protective behavioral strategies. In a large trial, we found that young adults exposed to the first TRAC intervention, TRAC1, reported greater reductions in alcohol consumption and alcohol-related injuries compared with control and assessment-only groups up to 6 months after intervention completion [6]. Still, effects of TRAC1 were small, indicating the intervention was not optimized.

One design feature potentially limiting TRAC1’s effectiveness was that drinking limit goal support was not optimized. In examining EMA data collected from those exposed to TRAC1, we found that subjects declined to commit to a weekend drinking limit goal based on the binge threshold roughly 40% of the times they were prompted and that goals were met only 65% of the time [7]. In examining latent classes of individuals exposed to TRAC1, we found that the class with higher baseline drinking had lower probability of committing to weekend drinking limit goals and had no discernible reduction in drinking over time [8]. Finally, in focus groups, several TRAC1 subjects reported ignoring goal prompts or declined goal commitment because they felt consuming <4 or 5 drinks was unreasonable based on the amount they drank, which was typically >10 drinks [9]. Together, these results provided evidence that design features focused on personalizing goal support, especially for heavier drinkers, needed to be improved.

To improve context-specific goal support, we incorporated drinking limit goal commitment EMA tailored to past 2-week alcohol consumption, intraweekend goal reminders, self-efficacy EMA with support tailored to goal confidence, and maximum weekend alcohol consumption EMA with drinking limit goal feedback into the TRAC intervention (now called TRAC2).

Our primary aim was to evaluate young adult engagement with the TRAC2 goal support features. Specifically, we examined responses to goal commitment and goal self-efficacy EMA, and how responses related to event-level alcohol consumption. We hypothesized that the percentage of responses indicating a willingness to commit to a weekend drinking limit goal tailored to past drinking (adaptive goal prompts) would be higher compared with the 60% willing to commit to the fixed binge threshold used in TRAC1. Our secondary aim was to examine the association of length of TRAC2 exposure with alcohol-related outcomes from baseline to 3-month follow-up, including maximum drinks per drinking occasion on weekends, prevalence of reporting a binge drinking episode on a typical drinking week, and alcohol-related consequences. We hypothesized that subjects who used the TRAC2 intervention for longer periods would have greater reductions in alcohol-related outcomes than those who used it for shorter periods.

## Methods

### Procedures

#### Recruitment and Enrollment in the Emergency Department

**Figure 1:**
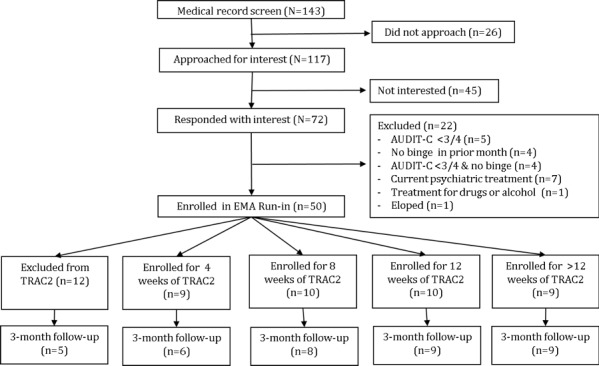
CONSORT diagram (EMA, ecological momentary assessments; AUDIT-C, Alcohol Use Disorder Identification Test for Consumption; TRAC2, Texting to Reduce Alcohol Consumption 2).

From April 1 to June 9, 2016, a convenience sample of 143 patients aged 18 to 25 years who presented to an urban emergency department (ED) were identified through medical record review. Following introduction by a member of the care team, 117 young adults who were medically stable and not seeking treatment for substance use disorder were approached by research staff and 72 (66.7%) of them provided consent to complete a questionnaire, including assessments of alcohol use severity. Young adults reporting recent hazardous alcohol consumption based on an Alcohol Use Disorder Identification Test for Consumption (AUDIT-C) score of ≥3 for women or ≥4 for men [9] and at least 1 binge drinking episode in the prior month were eligible to participate. Young adults were excluded if they reported past treatment for drug or alcohol use, reported current medical treatment for psychiatric disorders, or did not own a mobile phone with SMS. Excluded young adults (n=22) did not differ in age or sex from those included (n=50). All subjects provided written informed consent and completed a baseline survey in the ED, for which they were compensated US $20. All procedures were approved by the university’s institutional review board. The flow diagram of subject recruitment, enrollment, and retention is shown in Figure 1.

#### Text Message Run-In

Following enrollment (n=50), each Thursday at 5 pm for 2 weeks, subjects received the following EMA: “Do you plan on drinking this weekend?” Each Sunday at 1 pm for 2 weeks they received: “Between Thursday and today, what is the MOST drinks you had on any occasion?” When a subject responded, he or she received a text: “Thanks for completing this assessment. We will check in with you on [Thursday and Sunday].” If subjects completed at least 50% of EMA during the 2-week run-in, they were offered the option of enrolling in the TRAC2 intervention by texting “Go.” We included the run-in period to exclude those individuals who expressed initial willingness in participation but did not exhibit adequate engagement through texting.

#### Texting to Reduce Alcohol Consumption 2 Intervention

Subjects who met the run-in eligibility criteria and opted in (n=38) were sent EMA each Thursday, Friday, Saturday, and Sunday. On Thursdays, subjects were queried about their weekend drinking plans and willingness to commit to a drinking limit goal. Instead of asking individuals to commit to a weekend drinking limit goal based on the binge threshold (as done in TRAC1), we used an algorithm that prompted a drinking limit goal based on the running average of the largest number of drinks consumed by that individual on any occasion in the prior 2 weekends. When the average number of drinks in prior 2 weeks was greater than 10, the drinking limit goal was set at 10. When the average number of drinks in prior 2 weeks (#) was reported as less than or equal to 10 but greater than binge (>4/5 drinks), their drinking limit goal was (#) minus 1. This adaptive goal prompt feature fits with harm reduction principles [10] to meet individuals “where they are at” and the theory of behavioral shaping, where individuals with higher drinking amounts make small, successive approximations to an ultimate low-risk drinking goal (eg, binge threshold) [11].

To increase goal salience proximal to contexts of high cue reactivity and peer pressures [12], we sent goal reminders on Friday and Saturday evening (if they had committed to a drinking limit goal). We also queried their confidence in meeting this goal and tailored self-efficacy support based on their reply. This feature fits with prior research showing the importance of self-efficacy as a predictor of heavy drinking [13]. On Sundays, we queried the maximum number of drinks consumed on any occasion and, based on whether they committed to a goal, sent goal-relevant feedback (success or failure reframing) [14] or feedback on amount consumed.

In addition to these goal support features, we chose to provide individuals more control over how long they use the TRAC2 intervention using enhanced active choice [15], where at the end of each 4-week block, individuals were offered the choice to opt in for continued voluntary enrollment. Following each 4-week block, all existing TRAC2-enrolled subjects were given the opportunity to re-enroll by texting “Go,” up to a maximum of six 4-week blocks. A flow diagram of the TRAC2 intervention is provided in Multimedia Appendix 1.

#### Web-Based Follow-Up

All subjects, including those enrolled in the ED but not exposed to TRAC2, were asked to complete a follow-up Web-based survey 3 months after baseline assessments and were compensated US $40 upon completion. Subjects were notified by SMS to access the survey website and those who did not complete their Web-based follow-up within 1 week were contacted once through email as a reminder.

### Measures

#### Demographic Characteristics

At baseline, subjects reported their age, sex, race, ethnicity, and education (college enrolled: yes/no).

#### Alcohol-Related Characteristics

During screening, we asked subjects the AUDIT-C and the question: “How many days have you had ≥4 (for women) or ≥5 (for men) standard alcoholic drinks in the past month?” At baseline and at 3-month follow-up, subjects were presented with a calendar and a visual reference displaying standard drink amounts and were asked to report their alcohol consumption by day of the week for both a typical and heavy drinking week (Daily Drinking Questionnaire [16]). We used data from the heavy drinking week to calculate the maximum drinks consumed over any weekend day, and we used data from the typical week to calculate the percentage of subjects reporting any binge drinking episode. The 24-item Brief Young Adult Alcohol Consequences Questionnaire [17] was used to assess the number of negative alcohol-related consequences experienced during the past month. Items were dichotomous (no/yes) and summed. We used the Alcohol Ladder [18] to measure an individual’s motivation to change their drinking. The Alcohol Ladder is a visual analog scale that consists of 10 rungs, each with a corresponding statement (eg, “I never think about changing the way I drink, and I have no plans to change”). We coded responses to fit the stage of change [19] continuum (precontemplation, contemplation, preparation, action, and maintenance).

#### Drug Use

At baseline, subjects were asked to report frequency of other drug use over the past 3 months using the NIDA Modified Alcohol, Smoking, and Substance Involvement Screening Test (NM-ASSIST [20]). Cigarette use was recoded as less than daily=0 and at least daily use=1. Marijuana use and opioid use were recoded into dichotomous variables (none=0; any=1).

#### Ecological Momentary Assessments

Drinking intentions were measured each Thursday through responses to: “Do you plan on drinking this weekend?” coded as no=0 and yes=1. Willingness to commit to a drinking limit goal was measured through responses to: “Would you be willing to commit to a goal to drink less than [X] drinks on any occasion this weekend?” coded as no=0; and yes=1. Drinking limit goal self-efficacy was measured through responses to: “How confident are you that you will meet this goal on a scale from 1 (not at all) to 5 (completely)?” For the purposes of this study, we used the lowest value reported over the weekend as a measure of self-efficacy vulnerability. Finally, we measured the maximum number of drinks consumed each weekend through responses to: “Between Thursday and today, what is the MOST drinks you had on any occasion? *”* reported as a continuous variable. We used this value to calculate whether they met their drinking limit goal (when made), coded as did not meet goal=0 and met goal=1.

#### Data Analyses

We first examined baseline characteristics of enrolled subjects, identifying any differences across groups of different durations of TRAC2 engagement (number of 4-week blocks enrolled) using analysis for variance for mean comparisons, Kruskal-Wallis test for medians, and chi-square test for categories. We then calculated EMA response rates across weeks by group. To understand changes in alcohol consumption over weekends, we visually inspected distribution of maximum drinks consumed on any weekend day across groups and modeled data by group using repeated-measures linear regressions. We first declared data as a panel to account for clustering within individuals. We specified “max drinks” as a count variable with a Poisson distribution and modeled random effects, given the variability in maximum drinks consumed by subjects in week 1 (intercept). We specified that residuals were autoregressive. To understand predictors and processes influencing goal success, we used chi-square tests to examine univariate associations between selected correlates of drinking behavior and drinking goals being met. Finally, we tested the significance of differences between baseline and 3-month follow-up reports using Wilcoxon signed-rank tests (for maximum drinks and negative consequences) and 2-sample test of proportions (for prevalence of any binge drinking episode in a typical week). All statistical tests were conducted using Stata 14.0 (StataCorp, Inc, College Station, TX).

## Results

### Texting to Reduce Alcohol Consumption Subjects

A total of 50 subjects were enrolled in the ED. Baseline descriptive statistics are reported in Multimedia Appendix 2. There was a wide range of drinking severities, with 12% (6/50) of subjects scoring ≥10 on the AUDIT-C, indicating high probability of alcohol dependence [21]. There was also a wide range of stages of change, with 38% (9/50) of subjects being precontemplative. All subjects reported at least one negative consequence related to alcohol consumption in the last 3 months (median=9; interquartile range= 4-12). Substance use was common: around a quarter (26%, 13/50) of subjects smoked cigarettes at least daily, half (50%, 25/50) reported cannabis use, and 10% (5/50) used some form of opioid recreationally in the past month.

### Texting to Reduce Alcohol Consumption 2 Engagement

#### Opting in to Texting to Reduce Alcohol Consumption 2 Over Six 4-Week Intervention Blocks

Among the 50 enrolled subjects, 38 (76%) completed the run-in successfully and enrolled in TRAC2. Comparing those who were excluded during the run-in with those who successfully enrolled in the intervention period, there was a higher percentage of Hispanic subjects (25% [3/12] vs 8% [1/38]; *P*=.01) and lower percentage of college-enrolled subjects (8% [1/12] vs 32% [12/38]; *P*=.12). The percentage of subjects who continued to enroll in TRAC2 was 76% (29/38) after the first 4-week block, 50%(19/38) after the second 4-week block, 24% (9/38) after the third 4-week block, 16% (6/38) after the fourth 4-week block, 5% (2/38) after the fifth 4-week block, and 2% (1/38) after the sixth 4-week block. The only significant differences in baseline characteristics between subjects based on length of TRAC2 enrollment were higher prevalence of cannabis use among subjects enrolled for 4 weeks and higher prevalence of opioid use among those enrolled for 12 weeks. Stage of change was not associated with length of voluntary TRAC2 enrollment (see Multimedia Appendix 2).

#### Ecological Momentary Assessment Compliance Over the First 12 Weeks

**Figure 2:**
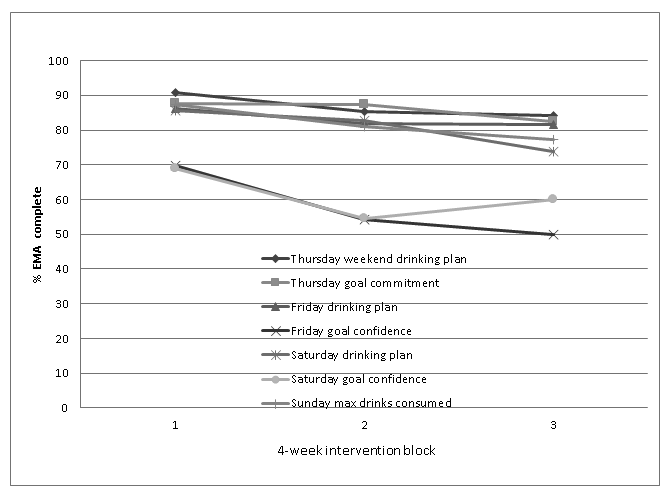
Ecological momentary assessment (EMA) completion rates by intervention block (TRAC2, Texting to Reduce Alcohol Consumption 2; CIs and/or standard errors are not included to allow for clarity).

The percentage of subjects responding to EMA queries on Thursday and Sunday by length of TRAC2 enrollment is shown in Figure 2. Response rates to EMA queries were, on average, 82.3% for the first 4-week intervention block, 75.3% for the second 4-week block, and 72.8% for the third 4-week block. Those subjects who opted in to TRAC2 for longer periods also had higher overall EMA completion rates. Response rates were lowest on Friday and Saturday evenings to goal self-efficacy EMA, where only 58% of EMA were responded to.

### Changes in Drinking-Related Outcomes

#### Ecological Momentary Assessment: Drinking Cognitions

In week 1, 78% (29/37) of subjects reported a plan to drink over the weekend, which decreased to 46% (15/33) by week 4. Among subjects who reported a plan to drink over a given weekend in the first 4 weeks of the intervention, on average reported being willing to commit to the proposed drinking limit goal 96% (68/71) of weekends. The percentage of subjects being prompted to commit to a drinking limit goal above the binge threshold was 52% (15/29) in week 1 and decreased to 0% (0/15) by week 4. The percentage of weekend days where subjects reported high confidence (score 4 or 5) in meeting their drinking limit goals on both Friday and Saturday in the first 4 weeks of the intervention was, on average, 60% (68/71 weekends), with no changes in confidence across time.

#### Ecological Momentary Assessment: Weekend Drinking and Goal Success

**Figure 3:**
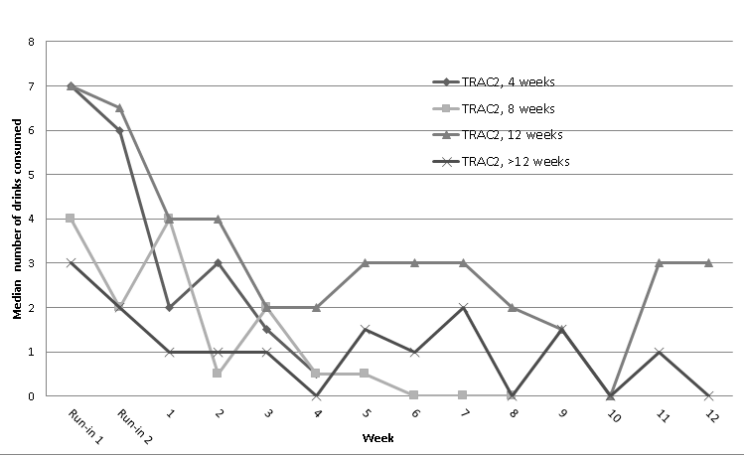
Median maximum drinks consumed over weekends by length of intervention engagement. Included are ecological momentary assessment (EMA) reports over the 2-week run-in to show changes that occurred before Texting to Reduce Alcohol Consumption 2 (TRAC2) intervention exposure (assessment reactivity).

The median number of maximum drinks consumed on any weekend day by length of TRAC2 enrollment and regression model output is shown in Figure 3 and Table 1. In Poisson regressions, all groups, except for those enrolled >12 weeks, significantly reduced their drinking over time. However, those enrolled >12 weeks had a lower starting point (intercept) for maximum drink count.

**Table 1:** Output from Poisson repeated-measures regression models of maximum drinks consumed over weekends using ecological momentary assessment (EMA) reports. Coefficient=beta coefficient.

TRAC2		Coefficient	95% CI	*P* value
**4 weeks (n=6)**				
	Intercept	2.82	2.18 to 3.46	<.001
	Rate of Change	–0.34	–0.47 to –0.21	<.001
**8 weeks (n=8)**					
	Intercept	1.6	0.87 to 2.32	<.001
	Rate of Change	0.17	–0.26 to –0.09	<.001
**12 weeks (n=9)**					
	Intercept	1.99	1.58 to 2.4	<.001
	Rate of Change	–0.13	–0.16 to –0.09	<.001
**>12 weeks (n=9)**					
	Intercept	0.61	0 to 1.21	.05
	Rate of Change	–0.03	–0.06 to –0.01	.10

**Table 2:** Change in alcohol-related outcomes from baseline to 3-month follow-up (Wilcoxon signed-rank tests for max drinks and negative consequences and 2-sample test of proportions for prevalence of any binge drinking episode in a typical week). Text in italics represents a summary of the other categories.

Outcome and TRAC2^a^ exposure		Baseline	3 months	*P* value
**Maximum drinks on any weekend day, median (IQR^b^)**					
	None (n=5)	6 (2-8)	9 (2-10)	.89
	4 weeks (n=6)	5 (3-10)	4 (2-7)	.29
	8 weeks (n=8)	5 (3-7)	5 (3-7)	.94
	12 weeks (n=9)	5 (4-6)	5 (3-8)	.6
	>12 weeks (n=9)	6 (3-6)	2 (1-5)	.09
	*Any (n=32)*	*5 (3-6)*	*5 (2-7)*	*.31*
**Any binge drinking episode in a typical week, n (%)**					
	None (n=5)	2 (40)	2 (40)	.99
	4 weeks (n=6)	4 (67)	2 (33)	.32
	8 weeks (n=8)	3 (38)	2 (25)	.56
	12 weeks (n=9)	3 (33)	4 (44)	.65
	>12 weeks (n=9)	4 (44)	1 (11)	.08
	*Any (n=32)*	*14 (44)*	*9 (28)*	*.19*
**Number of negative consequences, median (IQR)**					
	None (n=5)	9 (3-9)	3 (0-9)	.41
	4 weeks (n=6)	11 (3-14)	5 (1-7)	.4
	8 weeks (n=8)	9 (4-13)	2 (0-3)	.09
	12 weeks (n=9)	10 (4-13)	3 (1-6)	.009
	>12 weeks (n=9)	6 (4-11)	2 (0-3)	.05
	*Any (n=32)*	*9 (4-13)*	*3 (0-6)*	*.004*

^a^TRAC2: Texting to Reduce Alcohol Consumption 2.

^b^IQR: interquartile range.

The percentage of weekends where the drinking limit goal was met was, on average, 89.0% (130/146 weekends), with no significant change over time. When examining factors associated with goal success, we found that goal success rates were higher among black subjects (98% [51/52 weekends]) than white subjects (84% [58/69 weekends]; *P*=.01). There were lower rates of meeting drinking limit goals when the goal prompt was greater than binge levels (77% [26/34 weekends]) than when the goal was at the binge threshold (92.9% [104/112 weekends]; *P*=.01). Finally, there were lower rates of goal success when subjects reported lower confidence (score <4) in meeting the goal on either Friday or Saturday (76% [32/42 weekends]) compared with that when subjects reported high confidence (98% [56/57 weekends]; *P*=.001).

### Web-Based Retrospective Reports

A total of 37 out of 50 subjects (n=32 exposed; n=5 excluded from TRAC2) completed Web-based follow-up surveys at 3 months. No baseline factors including sex, college education, race, baseline alcohol use severity (AUDIT-C score), or stage of change were associated with attrition. There were trends indicating reductions in maximum drinks consumed over typical weekends and prevalence of binge drinking in all groups exposed to TRAC2. There were significant reductions in the number of alcohol-related consequences among TRAC2-exposed participants (see Table 2).

## Discussion

### Main Findings

In this study, we found high levels of engagement with a text message intervention incorporating adaptive goal support features among a racially diverse sample of nontreatment-seeking young adults at varying stages of change. Among subjects who met run-in criteria, there were high response rates to EMA during TRAC2 exposure. Consistent with our a priori hypothesis, we found that there was a high willingness to commit to adaptive drinking limit goals (96% of time), which is higher than the 40% goal commitment willingness when we used a fixed “binge” threshold with a similar cohort of young adults in TRAC1 [7]. This suggests that individuals find that goals for limiting drinks close to, but less than, typical drinking amounts are found to be more palatable than drinking limits that require larger reductions from typical drinking amounts. We also found that the proportion of weekends when goals were met was significantly higher than that found in TRAC1. This is likely due to the smaller “step-down” for each week, and could reduce the possibility of “limit violations,” which can be detrimental to future self-regulation of behavior [22]. Finally, we found that lower confidence in meeting drinking limit goals was associated with a lower probability of goal success. This finding supports the role of self-efficacy in drinking self-regulation [23].

Regarding the safety of prompting individuals to commit to goals to limit drinks at levels found to be associated with negative outcomes, we found that it was a time-limited issue. Despite more than half of subjects being prompted in week 1 to commit to a drinking limit goal above a binge threshold, by week 4 no subject was being prompted to limit drinks above binge threshold. We also recognize that the binge threshold is a somewhat arbitrary cutoff and that there is a linear relationship with escalating blood alcohol content and consequences [24], thus supporting goal prompts that work to assist any reduction in alcohol consumption from typical amounts.

To our knowledge, this is the first published report of an alcohol intervention that incorporates an algorithm that gradually steps an individual down gradually over time. We focused on goal support, given the importance of goals in behavior change generally [25] and for self-regulation of substance use among young adults specifically [26]. Although there have been no prior alcohol interventions that use adaptive goal prompts, behavioral studies outside the alcohol field have used adaptive goal algorithms to improve step counts among obese adults [27] and reduce smoking by using a criterion based on percentile carbon monoxide levels [28].

### Secondary Findings

We found that 76% (29/38) of enrolled subjects chose to continue the TRAC2 program after the first 4 weeks. We were surprised that a third of subjects chose to continue the program after 12 weeks, with 1 individual continuing the program up to 28 weeks. These findings suggest that most individuals exposed to TRAC2 find it valuable. It also highlights the importance of choice architecture in behavioral intervention designs and supports “enhanced active choice” [15] where users control length of participation. We did not find that subjects who used the TRAC2 intervention for longer periods had greater reductions in drinking than those who used it for shorter periods. This may be due to the fact that those who used TRAC2 for longer periods had lower alcohol consumption at the start of the intervention, as evidenced through EMA reports in the >12 week group. It may also be that those individuals who did not re-enroll in TRAC2 had made desired reductions in their drinking over a short period and did not need further support, as evidenced through the rapid reductions in weekend maximum drink reports seen in the 4-week group. If this is true, then not all who drop out of mobile behavioral interventions should be considered “failures.”

### Limitations

This was a pilot study with a small number of participants. As such, differences may exist that we were not powered to detect. Subjects were sampled from an urban ED and therefore may not represent young adults broadly. All outcome data were self-reported and subject to possible bias. A quarter of young adults who expressed interest in study participation in the ED did not complete at least half of EMA sent over the first 2 weeks and were thus excluded from receiving the TRAC intervention. This may indicate that not all young adults with hazardous alcohol use are willing or interested in interacting through digital modalities such as SMS to improve health behaviors. We did not use randomization procedures, given the primary aim of determining acceptability, and therefore, cohorts may differ in both measured and unmeasured ways. Finally, the response rates to goal self-efficacy EMA were significantly lower than other EMAs. This may have been due to the fact that either the timing of the messages was not optimal (eg, received during socializing times) or the nature of the query was unacceptable.

### Conclusions

Preliminary evidence suggests that, among a diverse sample of nontreatment-seeking young adults with past hazardous alcohol consumption, adaptive goal support text message intervention features are acceptable and potentially effective in supporting short-term reductions in alcohol consumption. Future research is needed to replicate findings in a larger cohort and determine which features of adaptive goal support optimize behavioral change.

## Appendix

Multimedia Appendix 1 Flow diagram of Texting to Reduce Alcohol Consumption-2 (TRAC2).

Multimedia Appendix 2 Baseline characteristics by length of enrollment.

## Abbreviations

AUDIT-C: Alcohol Use Disorder Identification Test for Consumption
ED: emergency department
EMA: ecological momentary assessment
TRAC: Texting to Reduce Alcohol Consumption
