# Supplementary material for: A Text Message Intervention with Adaptive Goal Support to Reduce Alcohol Consumption Among Non-Treatment-Seeking Young Adults: Non-Randomized Clinical Trial with Voluntary Length of Enrollment
Source: JMIR Mhealth Uhealth. 2018 Feb 16;6(2):e35. doi: 10.2196/mhealth.8530 (PMC5834751; doi:10.2196/mhealth.8530)
Supplement: Multimedia Appendix 1 [file mhealth_v6i2e35_app1.pdf]

## Appendix

### Thursday 5pm: Pre-weekend Drinking Plans

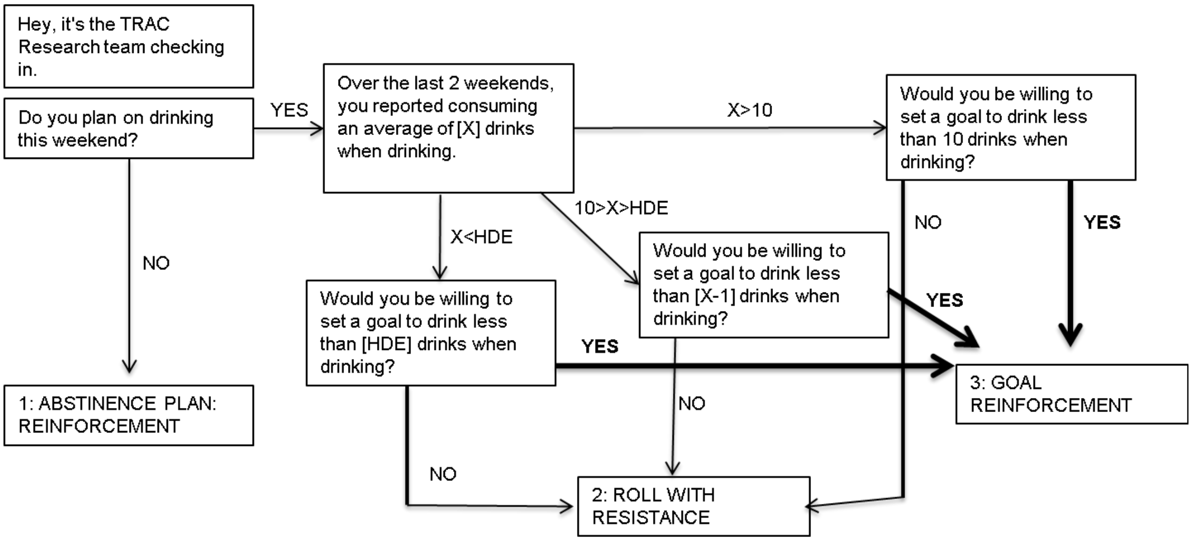


### Friday 5pm and Saturday 1pm: Weekend Drinking Plans

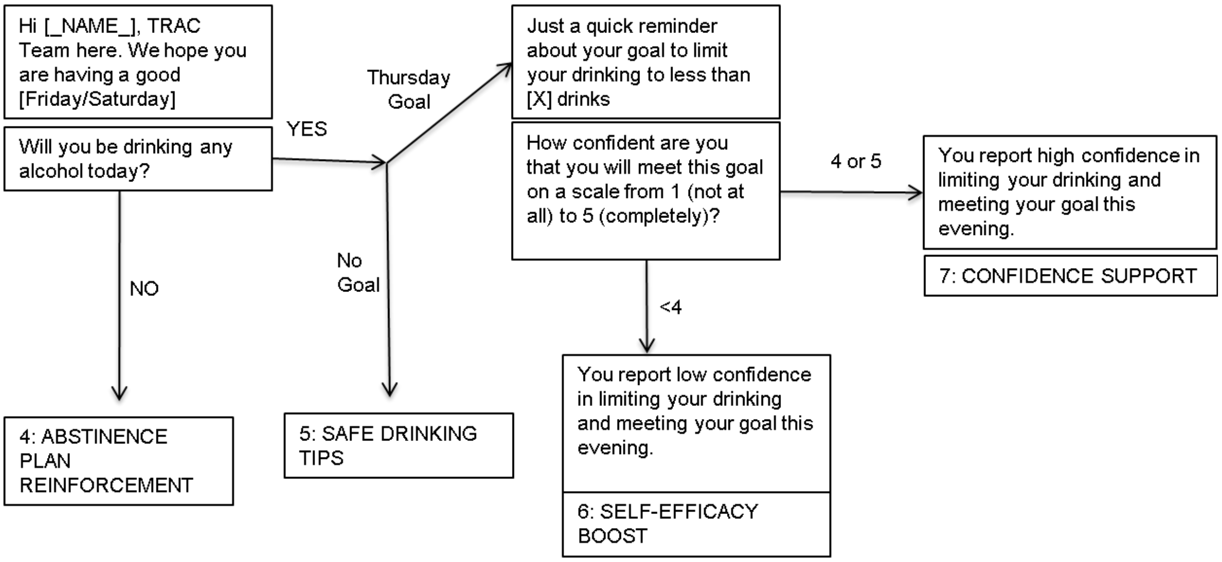

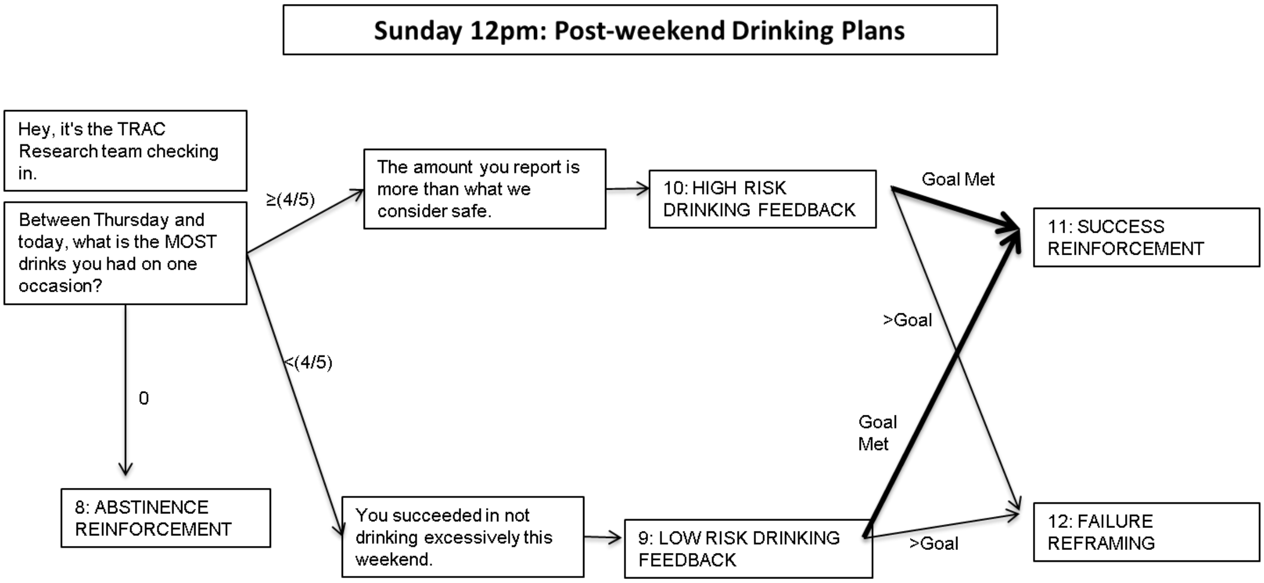
Sunday 12pm: Post-weekend Drinking Plans
Hey, it's the TRAC Research team checking in.
Between Thursday and today, what is the MOST drinks you had on one occasion?
≥(4/5)
<(4/5)
0
The amount you report is more than what we consider safe.
10: HIGH RISK DRINKING FEEDBACK
You succeeded in not drinking excessively this weekend.
9: LOW RISK DRINKING FEEDBACK
8: ABSTINENCE REINFORCEMENT
Goal Met
>Goal
Goal Met
>Goal
11: SUCCESS REINFORCEMENT
12: FAILURE REFRAMING
