# Supplementary material for: A Text Message Intervention with Adaptive Goal Support to Reduce Alcohol Consumption Among Non-Treatment-Seeking Young Adults: Non-Randomized Clinical Trial with Voluntary Length of Enrollment
Source: JMIR Mhealth Uhealth. 2018 Feb 16;6(2):e35. doi: 10.2196/mhealth.8530 (PMC5834751; doi:10.2196/mhealth.8530)
Supplement: Multimedia Appendix 2 [file mhealth_v6i2e35_app2.pdf]

Baseline characteristics by length of enrollment (analysis of variance for mean comparisons, Kruskal-Wallis test for medians, and chi-square test for categories).

Characteristics		Enrolled in EMA[a] run-in (n=50)	TRAC2[b] excluded (n=12)	TRAC2, 4 weeks (n=9)	TRAC2, 8 weeks (n=10)	TRAC2, 12 weeks (n=10)	TRAC2, >12 weeks (n=9)	*P* value
Age, mean (SD)		22 (1.8)	23.5 (1.7)	22.9 (1.7)	23.2 (1.9)	22.6 (1.7)	23.7 (2.1)	.69
Female, n (%)		28 (56)	6 (50)	4 (44)	4 (40)	5 (50)	3 (33)	.63
**Race, n (%)**								
	White/Caucasian	23 (46)	7 (58)	4 (44)	2 (20)	6 (60)	4 (44)	.37
	African American	27 (54)	5 (42)	5 (56)	8 (80)	4 (40)	5 (56)	
Hispanic ethnicity, n (%)		4 (8)	3 (25)	0	0	0	1 (11)	.12
Current college enrollment, n (%)		12 (32)	1 (8)	3 (33)	3 (30)	3 (30)	3 (33)	.62
**Baseline alcohol use**								
	AUDIT-C[c] score, median (IQR[d])	7 (5-9)	8 (5-9)	8 (7-10)	7 (5-9)	6.5 (4-7)	7 (6-7)	.44
	Maximum drinks on any weekend day, median (IQR)	5 (3-6)	6 (2-9)	5 (4-10)	4 (2-5)	5 (4-6)	6 (3-6)	.63
	Any binge drinking in typical week, n (%)	25 (50)	8 (67)	6 (67)	4 (40)	3 (30)	4 (44)	.35
	Number of negative consequences, median (IQR)	9 (4-12)	10 (5-11)	8 (3-13)	9 (5-10)	9 (4-13)	6 (4-11)	.99
**Stage of change, n (%)**								
	Precontemplative	19 (38)	5 (41)	3 (33)	4 (40)	2 (20)	5 (62)	.85
	Contemplative	13 (26)	3 (25)	3 (33)	3 (30)	3 (30)	0	
	Preparation	8 (16)	2 (16)	0	1 (10)	3 (30)	2 (25)	

	Action or maintenance	10 (20)	2 (16)	3 (33)	2 (20)	2 (20)	1 (13)	
**Other drug use, n (%)**								
	At least daily tobacco	13 (26)	1 (8)	3 (33)	3 (30)	4 (40)	2 (22)	.50
	Any cannabis	25 (50)	5 (42)	8 (89)	6 (60)	2 (20)	4 (44)	.04
	Any opioid	5 (10)	0	1 (11)	0	4 (40)	0	.01

[a]EMA: ecological momentary assessment.

[b]TRAC2: Texting to Reduce Alcohol Consumption 2.

[c]AUDIT-C: Alcohol Use Disorder Identification Test for Consumption.

[d]IQR: interquartile range.
